# Urinary incontinence and its association with pelvic floor muscle exercise among pregnant women attending a primary care clinic in Selangor, Malaysia

**DOI:** 10.1371/journal.pone.0236140

**Published:** 2020-07-15

**Authors:** Aida Jaffar, Sherina Mohd-Sidik, Foo Chai Nien, Gan Quan Fu, Nor Hazlin Talib

**Affiliations:** 1 Primary Care Unit, Faculty of Medicine and Defence Health, Universiti Pertahanan Nasional Malaysia, Sg Besi, Wilayah Persekutuan Kuala Lumpur, Malaysia; 2 Department of Psychiatry, Faculty of Medicine and Health Sciences, Universiti Putra Malaysia, UPM Serdang, Selangor, Malaysia; 3 Department of Population Medicine, Universiti Tunku Abdul Rahman, Kajang, Selangor, Malaysia; 4 Pre-clinical Department, Universiti Tunku Abdul Rahman, Kajang, Selangor, Malaysia; 5 Klinik Kesihatan Kajang, Kajang, Selangor, Malaysia; University Medical Center Utrecht, NETHERLANDS

## Abstract

**Background:**

Urinary Incontinence (UI) is when a person is unable to hold his/her urine effectively. This is a common problem which can develop and worsen during pregnancy. An effective way to manage UI is to educate patients on the Pelvic Floor Muscle Exercise (PFME) regularly. The present study aimed to ascertain the pregnant women’s knowledge, attitudes, and practices (KAP) related to PFME.

**Methods:**

This was a cross-sectional study done in a one primary care clinic located in a semi-urban area in Selangor, Malaysia. Simple random sampling was conducted among pregnant women aged 18 years old and above at any gestation. The validated study instruments used consisted of questions on socio-demography, KAP on UI, and also the International Consultation on Incontinence Questionnaire-Urinary Incontinence Short Form to determine UI among the respondents.

**Results:**

The response rate for this study was 72.1%, where 440 pregnant women consented to take part in the study. The median age of study respondents was 30 years old and majority of the study respondents was from the Malay ethnicity (80.9%). The prevalence of UI was 40.9%. The proportion of pregnant women with good knowledge, attitude and practice scores were 58.0%, 46.6% and 45.2% respectively. There was a significant association between UI and age (p = .03), body mass index (p = .03), ethnicity (p = .04), gravida. (p = .001), knowledge on PFME (p = .007) and attitude towards PFME (p = .006).

**Conclusions:**

Findings from this study fill a gap in the prevalence and KAP concerning PFME at the primary care level. The foundation areas for future education and health promotion on UI should address the importance of correct PFME. This education can be delivered through a pragmatic way to ensure its effectiveness and sustainability of the health promotion program.

## Introduction

Urinary incontinence (UI) also known as the involuntary leakage of urine is one of the most common health problems affecting women globally. According to statistics, 423 million (21.6%) women were diagnosed with UI in 2018, as such it has become one of the greatest regional burdens in Asia [1]. Both individuals and their families experienced significant psychosocial impact as they have never discussed with a healthcare professional [2] and will gradually affect their quality of life if left untreated [3].

International Continence Society (ICS) classified the UI into different types including stress UI, urge UI and mixed UI [4]. Stress UI (SUI) is characterised by involuntary leakage on sneezing or coughing or exertion [4]. Urge UI (UUI) is leakage either immediately or accompanied by urgency [4]. Mixed UI (MUI) is involuntary leakage associated with urgency and with sneezing, coughing or exertion [4]. Pregnancy and especially vaginal delivery are the major causes of SUI. The functional support of pelvic floor muscles is impaired due to reduced total collagen content and its tensile properties, resulting in joint laxity [5, 6]. Statistics have reported where 17–54% of the female population experienced the first UI during pregnancy [7].

In tertiary centres itself, UI has been reported to increase considerably in accordance to the trimester of pregnancy in addition to the number of pregnancies [8]. In a Turkish study it was reported the third trimester participant had three times the risk of UI compared to the first trimester respondents Odds Ratio (OR = 3.206, 95% CI,1.1788, 0.725), p = .023 [9]. Similarly another study in Spain reported the prevalence of UI was 19% (11 of 58) in the first trimester and 39.8% (66 of 166) in the third trimester (p = 0.004) [10]. A recent local study done in a Patient Assessment Centre of a tertiary referral hospital in Klang Valley reported that UI among 306 antenatal women in their third trimester was 34.3% with the most prevalent type being SUI (64.8%) followed by MUI (24.8%) and UUI (6.7%) [11].

Regardless of the types of incontinence, studies have reported pelvic floor muscle exercise (PFME) to be an effective exercise in treating SUI and MUI by strengthening the pelvic floor muscles [12–16]. Many randomized controlled trials have reported positive results using PFME as a preventative measure and intervention in treating and preventing UI among pregnant and postpartum women [7, 17]. Hence, this exercise should be a standard component of perinatal care, and women should be advised and taught to perform PFME during pregnancy and postpartum.

In addition, only 37.2% of 101 pregnant women who attended PFME educational classes have better knowledge, attitude, and practice (KAP) towards PFME [18]. There are some adherence concerns where pregnant women are reluctant to attend a supervised PFME class especially women who were working / employed due to time constrain [19].

In addressing such issues, we selected antenatal women as our target population. Based on the above studies conducted in Malaysia, UI was found to occur commonly among pregnant women [11, 20]. However, none of the studies identified any preventative measures being used in primary care settings. As primary care is the gatekeeper where first line practitioners are responsible for the diagnosis, management and referral of medical cases, it is important that these healthcare professionals have adequate knowledge on PFME. Antenatal PFME educational classes in primary care settings would reduce the risk of UI among antenatal women. Therefore, this study was conducted to the determine the prevalence of UI among pregnant women in a primary care setting. We also determined the KAP towards PFME among these women, so that the findings could help us in planning effective preventive care strategies using PFME among pregnant women who are at risk of developing UI. The results of this study will serve as an input for policy decision-making to implement PFME in primary care settings with the aim of reducing the risk of UI in pregnant women. Reducing UI could help to save cost, burden, and time in managing complications arising from UI among women.

## Materials and methods

### Study design and selection of respondents

A cross-sectional study was conducted in one primary care clinic in Hulu Langat District, Selangor. Selangor is the most populated state in Malaysia with 244 government health clinics, where pregnant women come for their antenatal check-ups [21]. The study was conducted in accordance with the principles established by the Declaration of Helsinki, with informed consent obtained from the patients. A simple random sampling was applied to pregnant women who were 18 years old and above at any week of gestation with singleton pregnancy and no other medical or psychiatric illness. This data collection was done for four months in duration (September 2019 till December 2019). The inclusion criteria are pregnant mothers with a singleton pregnancy, any gravida, able to communicate and read in Malay or English. The exclusion criteria for this study are pregnant women those with a history of childhood enuresis or pelvic surgery or pelvic organ prolapse, history of mental illness or psychosis, and history of poorly controlled Diabetes Mellitus as the symptoms may similar with symptoms of UI.

Sample size calculation for this study was based on the formula by S.K. Lwanga and Lemeshow (1991), with the prevalence of UI among pregnant women at 65.8% [20], α = 0.05 and 95% power of study. The estimated sample size calculated was 350 [22]. After considering 20% for non-respondents, the final sample size was calculated to be 440.

### Data collection

Upon arrival at the antenatal clinic for a routine visit, all pregnant women met a staff nurse at the registration counter. The nurses determined the pregnant women’s eligibility for the study from each woman’s antenatal card. Simple random sampling was done to select patients who were eligible and informed about this study. They were asked if they would like to know more and participate in the study.

Further information about the study was provided before obtaining informed consent. Participants were given the questionnaires to be filed with the average of 15–30 minutes. They were accompanied by the research assistants to the usual clinic room for their antenatal care appointment.

However, if the pregnant women declined to participate, they proceeded with their antenatal care appointment as usual.

### Study instruments

The data instrument for this study was by self-administered questionnaire. The total duration to answer all the questionnaires took up to 30 minutes in duration. The questionnaire consisted of 2 main sections; (1) Knowledge, Attitude and Practice on PFME (KAP) and (2) the International Consultation on Incontinence Questionnaire-Urinary Incontinence Short Form (ICIQ-UI SF) which assessed the frequency of UI, amount of leakage, the overall impact of UI and diagnosis of the type of incontinence. In this study, the validated Malay version was used [23].

ICIQ-UI SF was highly recommended by the 2004 International Consultation on Incontinence, with a Grade A status on standard validation criteria [24]. ICIQ-UI SF can assess frequency and volume in diagnosing UI. From these questions, the minimum scores for ICIQ-UI SF is 3 and the maximum scores is 21 points. This study used the adapted severity scoring of slight (1–5), moderate (6–12), severe (13–18), and very severe (19–21) [25].

The KAP for PFME assessed the knowledge, attitude, and practices towards PFME has been locally developed and validated [26]. The Cronbach’s alpha for the KAP questionnaire was good; 0.949 for knowledge, 0.837 for attitude and 0.742 for practices [26]. The knowledge section has categorical responses of true, false and don’t know. The attitude section has 5 likert scales for its responses from strongly agree to strongly not agree. The practices section has categorical responses of never, seldom, frequent and always. For this study, the KAP questionnaire consisted of 26 items out of the original questionnaire of 29 items. Three items were removed due to sensitive issues in our culture with regards to sexual activities. The deleted items were from the Knowledge (K) part of the questionnaire. The mean of total scores was used to determine whether the respondents’ had good or poor knowledge, attitude and practise towards PFME [26].

Prior to this study, a small pilot study was done among thirty pregnant women to test the validity and reliability of the questionnaires. The Cronbach alpha coefficient was 0.622 for the ICIQ-UI SF. With regards to the KAP towards PFME, the Cronbach alpha was 0.925 for knowledge, 0.813 for attitude and 0.859 for practices.

The data were analysed using the Statistical Package for Social Sciences (SPSS) version 25.0 (SPSS Inc., Chicago, IL). The level of significance was set at a p-value of <0.05. Normality tests were performed prior to analysing the data for descriptive and inferential statistics.

Bivariate analysis using hi-square test was performed to see any significant associations between the independent and dependent variables. The predictors of urinary incontinence were determined using multiple logistic regression, after controlling for confounders. All variables with p<0.25 from the bivariate analysis were inserted into the multiple logistic regression model and the significant variables based on a p value of <0.05 were determined as predictors.

### Ethical consideration(s)

This study was approved by the Ethics Committee for Research Involving Human Subjects, Universiti Putra Malaysia (JKEUPM-2019-297) and the Medical Research and Ethics Committee (MREC), Ministry of Health Malaysia. The study is registered under the National Medical Research Register (NMRR-19-412-47116) and is funded under the Geran Putra Berimpak, Universiti Putra Malaysia (UPM) (UPM/800–3/3/1/GPB/2018/9668500). The study protocol is available.

## Results

The respondents for this study was 440 pregnant women with the response rate of 72.1%. About 170 pregnant women did not fulifll the criteria and refuse to join the study. The mean age was 29.8 years old (SD 4.69) and BMI was 24.5 kgm^2^ (SD 3.5). The majority of respondents were from the Malay ethnicity (80.9%), and from the low socio-economic status (81.9%) where the median financial income was RM4000 per month. The financial income was based on the country’s Gross Domestic Product (GDP), which was divided into B40, M40 and T20 critera [21]. B40 (Below 40%) consists of those with monthly income between RM3000 –RM6274, M40 (Middle 40%) consists of those with monthly income between RM6275-RM13147 and T20 (Top 20%) consists of those with monthly income at RM13148 and above [21].

About one-third (38.6%) of the respondents were primigravida, and 30.1% had a history of caesarean section among the multi-gravida respondents. About half the respondents (53.4%) were in their third trimester.

With regards to PFME knowledge, 58.0% had good knowledge. Less than half of them (46.6%) had good attitude and good practice (45.2%) towards PFME.

There was a significant association between UI and age (p = 0.03), BMI (p = .03), ethnicity (p = .03), gravida (p = .001), knowledge (p = .007) and attitude (p = .006) towards PFME as shown in Table 1.

**Table 1:** Demographics of the study respondents (N = 440).

Variables	No UI n (%)	UI n (%)	X^2^	P
Age			4.69	.03
Less than 30	138 (53.3)	77 (42.8)		
30 and above	121 (46.7)	103 (57.2)		
Education			1.15	.291
Low	174 (66.9)	129 (71.7)		
High	86 (33.1)	51 (28.3)		
Ethnicity			4.26	.039
Malay	202 (77.7)	154 (85.6)		
Non-Malay	58 (22.3)	26 (14.4)		
Income			0.30	.58
<RM3000	179 (82.9)	125 (80.6)		
≥RM3000	37 (17.1)	30 (19.4)		
BMI*			6.96	.031
Normal and below	123 (47.3)	63 (35.0)		
Overwieght	80 (30.8)	64 (35.6)		
Obese	57 (21.9)	53 (29.4)		
Gravida			15.22	.001
1	120 (46.2)	50 (27.8)		
2–5	120 (46.2)	110 (61.1)		
>5	20 (7.7)	20 (11.1)		
Trimester			3.96	.142
First	38 (14.6)	15 (8.3)		
Second	87 (33.5)	65 (36.1)		
Third	135 (51.9)	100 (55.6)		
Caesarean section			0.15	.698
No	101 (72.1)	91 (70.0)		
Yes	39 (27.9)	39 (30.0)		
Knowledge on PFME			7.22	.007
Poor	123 (47.3)	62 (34.4)		
Good	137 (52.7)	118 (65.6)		
Attitude towards PFME			7.55	.006
Poor	153 (58.8)	82 (44.6)		
Good	107 (41.2)	98 (54.4)		
Practice of PFME			1.19	.276
Poor	148 (56.9)	93 (51.7)		
Good	112 (43.1)	87 (48.3)		

# Financial status according to our GDP (21).

* BMI—Body Mass Index.

About 40.9% of the respondents had UI during pregnancy, and in terms of severity, most of them had slight UI with the mean score ICIQ-UI SF of 2.35 (SD 3.15). Only 1.1% experienced severe UI during pregnancy. UI occured mainly in the second and third trimester (Table 2).

**Table 2:** Urinary incontinence according the pregnancy trimester.

	Category Urinary Incontinence (UI) n (%)			
Trimester	No UI	Slight UI	Moderate UI	Severe UI
1	38 (14.6)	8 (8.4)	7 (8.8)	0
2	87 (33.5)	27 (28.4)	35 (43.8)	3 (60.0)
3	135 (51.9)	60 (63.2)	38 (47.5)	2 (40.0)
Total	260	95	80	5

The number of respondents who had SUI (answered “Yes” to both questions “urine leaks when cough or sneeze” and “urine leaks when physically active/exercising”) were 136 (30.9%) as shown in Table 3. This means that almost three-quarters (75.6%) of the respondents who had UI in this study were having SUI.

**Table 3:** Types of urinary icontinence using ICIQ-SF.

When does urine leak?	No n (%)	Yes n (%)
Never, urine never leak	177 (40.2)	263 (59.8)
Leaks before able to get to the toilet	398 (90.5)	42 (9.5)
Leaks when cough or sneeze	293 (66.6)	147 (33.4)
Leaks when sleep	434 (98.6)	6 (1.4)
Leaks when physically active/exercising	427 (97.0)	13 (13.0)
Leaks when finished urinating and are dressed	415 (94.5)	25 (5.7)
Leaks for no obvious reason	432 (98.2)	8 (1.8)
Leaks all the time	438 (99.5)	2 (0.5)

In terms of knowledge, most of the respondents did not know the correct way of doing PFME (three times a day, with 8 seconds minimum and 8–10 seconds each times of exercise). In terms of attitude, a majority agreed they should practice PFME to prevent UI and will make the effort to find information on PFME. In terms of practise, about a third (38.9%) had never done PFME, while more than half (57.0%) have never discussed PFME with friends. (Tables 4–6).

**Table 4:** Knowledge on PFME among pregnant women.

Knowledge on PFME	Don’t know n (%)	False n (%)	True n (%)
PFME muscles are situated at pubic region	168 (38.2)	44 (10.0)	228 (51.8)
PFME invloves muscles at anal region	187 (42.5)	57 (13.0)	196 (44.5)
Vagina muscles are not involve in PFME	157 (35.7)	234 (53.2)	49 (11.1)
PFM are important in controlling bladder function	136 (30.9)	7 (1.6)	296 (67.3)
PFM not involved in controlling anus	240 (54.5)	120 (27.3)	80 (18.2)
PFM not involved in tightening vagina	162 (36.8)	176 (40.0)	102 (23.2)
PFME can tighten buttocks muscles	207 (47.0)	40 (9.1)	193 (43.9)
PFME can prevent UI during laughing/sneezing/weight bearing	143 (32.5)	5 (1.1)	292 (66.4)
PFME can prevent/treat uterine prolapse	180 (40.9)	16 (3.6)	244 (55.5)
PFME can be done at any time	118 (26.8)	7 (1.6)	315 (71.6)
PFME can be done while performing daily activities	160 (36.4)	32 (7.3)	248 (56.4)
Muscles involved should be contracted for 8 seconds	248 (56.4)	8 (1.8)	184 (41.8)
PFM should be contracted 8–10 times per exercise	263 (59.8)	7 (1.6)	170 (38.6)
PFME should be done at least 3x a day (morning, afternoon,night)	271 (61.6)	11 (2.5)	158 (35.9)

PFM—Pevic floor muscle.

**Table 5:** Attitude towards PFME among pregnant women.

Attitude on PFME	Strongly disagree n (%)	Disagree n (%)	Neutral n (%)	Agree n (%)	Strongly Agree n (%)
PFME should be done by all women	8 (1.8)	26 (5.9)	196 (44.5)	169 (36.1)	45 (10.2)
I should practice PFME to prevent/treat UI	5 (1.1)	5 (1.1)	5.3 (12.0)	289 (65.7)	86 (19.5)
I should practice PFME to prevent uterine prolapse	8 (1.8)	21 (4.8)	88 (20.0)	249 (56.6)	72 (16.4)
I feel that PFME is boring	53 (12.0)	245 (55.7)	101 (23.0)	35 (8.0)	3 (0.7)
PFME should be taught to all antenatal mothers at antenatal clinics	9 (2.0)	10 (2.3)	53 (12.0)	256 (58.2)	111 (25.2)
I support those who want to perform PFME	7 (1.6)	5 (1.1)	44 (10.0)	270 (61.4)	113 (25.7)
I view that PFME can increase sexual satisfaction	5 (1.1)	17 (3.9)	122 (27.7)	220 (50.0)	73 (16.6)
I will put in effort to search for info about PFME	6 (1.4)	11 (2.5)	55 (12.5)	284 (64.5)	83 (18.9)

**Table 6:** PFME practice among pregnant women.

	PFME Practices	Never n (%)	Seldom n (%)	Usually n (%)	Frequent n (%)	Always n (%)
1	I have performed PFME when not pregnant	171 (38.9)	83 (18.9)	122 (22.7)	49 (11.1)	15 (3.4)
2	I have spent time to perform PFME	158 (35.9)	106 (24.1)	126 (28.6)	38 (8.6)	12 (12.7)
3	I have discussed PFME with friends	251 (57.0)	85 (19.3)	75 (17.0)	22 (5.0)	7 (1.6)
4	I have tried to search for info about PFME	167 (38.0)	91 (20.7)	128 (29.1)	42 (9.5)	12 (2.7)

Based on a p-value of 0.25, age, BMI, pregnancy category, ethnicity, knowledge and attitude towards PFME were entered into the multiple logistic regression analysis model to predict the UI among pregnant women. The Hosmer and Lemeshow test confirmed that the model was good fit for the data. As shown in Table 7, multigravida or gravida of 2–5 (OR 2.01, 95%CI 1.24–3.26) and obesity (OR 1.69, 95% CI 1.01–2.82) were significantly associated with increased odds for urinary incontinence.

**Table 7:** Predictors of urinary incontinence (N = 440).

	P	OR	95% CI
Gravida			
1	0.017	1	
2–5	0.004*	2.01	(1.24, 3.26)
>5	0.14	1.82	(0.82, 4.05)
Trimester			
First Trimester	0.239	1	
Second Trimester	0.138	1.72	(0.84, 3.53)
Third Trimester	0.093	1.81	(0.91, 3.60)
BMI			
Normal and low	0.105	1	
Overweight	0.142	1.43	(0.89, 2.29)
Obese	0.044*	1.69	(1.01, 2.82)
Knowledge on PFME			
Poor knowledge	0.363	1.82	(0.82, 4.05)
Attitude towards PFME			
Poor attitude	0.32	0.67	(0.30, 1.48)

UW-Underweight, BMI—Body Mass Index, OR—Odds Ratio, CI—Confidence interval.

## Discussion

UI is a condition present in some pregnant women at their first trimester which progresses more towards their third trimester. Based on this study’s findings, the prevalence of UI during pregnancy in Malaysia is relatively high where 40.9% of the respondents had UI. Similarly, another study investigated the prevalence of UI in a multi-ethnic population of pregnant women in Norway reported that the prevalence of UI among East Asian (40%) and South Asian (43%) were higher as compared to African (26%) and Middle Eastern populations (36%) [27]. The prevalence is only slightly lower as compared to the South American population (42%) [28].

The results showed that more than half of the respondents had good knowledge of PFME. Despite that, their attitude and practice on PFME were poor, indicating that the majority did not have the right attitude and practise towards PFME during pregnancy, although they knew the benefits and techniques of performing PFME. Lifestyle could be one of the possible contributing factors leading to poor practice of PFME among pregnant women. This may be due to the current trend in developing countries where women are busy multitasking as homemakers as well as working in various job sectors to improve their household income and also the country’s economy [29, 30]. Hence, due to the hectic lifestyle and time constrain, the practice of PFME during pregnancy is being neglected [19]. However, these are merely assumptions and future studies are required to identify the causes of poor practices towards PFME among pregnant women.

In Malaysia, studies on UI and PFME are mainly limited to studies conducted in tertiary care settings. So far, to our best knowledge and literature search, there are no studies conducted on PFME as an intervention or preventative measures for UI in primary care settings [31]. Therefore, this could be the first Malaysian study which examined the knowledge, attitude and practice of PFME among pregnant women in primary care settings.

This study also included different categories of pregnancy among the respondents; primigravida (38.6%), multigravida (52.3%) and grand multigravida (9.1%). The reason being that pregnant ladies who visited primary care settings are a mix of primigravida, multigravida and grand multigravida and are also in different trimesters. Thus, PFME in prevention and management of UI among pregnancy should be available to all pregnancy categories and trimesters. It had been reported that UI among primigravida was 34.4%, while most of the incontinence population were among the third trimester and suffering from SUI (64.8%). Our study also showed that the severity of incontinence increases throughout the trimesters. Therefore this study differs from two previous studies conducted in Malaysia, which focused on a single parity [31] and used a different type of questionnaire [32].

This study revealed that pregnant women who were obese had almost 2-fold higher risk to develop UI than those of normal weight. Similarly, a previous study also reported that pregnant women who were obese had a 9-fold higher risk of UI [Adjusted Odds Ratio [AOR] = 9.29, 95% CI (2.09, 41.37)]. As obesity is also one of the cause leading to UI, pregnancy with obesity could double up the risk of developing UI [33]. A cohort study on the other hand reported that rapid weight gain before week 15 of pregnancy was associated with UI at week 30 of pregnancy, while rapid weight gain after week 15 did not show such association [34].

Besides obesity, UI is also twice as common in multiparous women compared to nulliparous women. This is due to the structural pelvic changes which occurs among multiparous women after their first delivery. Such contexts have been proven from a study which compared the anatomical changes on the pelvic floor structure between nulliparous and multiparous women. The study reported that the Bladder Neck Decent (BND) was greater in multiparous than nulliparous women suggesting poor urethral support [35]. Similarly, another study also reported that the rate of UI in nulliparous women (31.8%) was lower than multiparous (47.9%) [35].

Based on local data, 96.4% of pregnant women who attended a tertiary center had positive attitude scores which were almost doubled as compared to this study’s findings [26]. Tertiary centers are probably more equipped in healthcare facilities as compared to primary care centers [36]. Hence, patients attending tertiary centers have more access to various supportive and preventative interventions [37]. Based on this study, 58.0% of respondents had good knowledge about PFME which is slighlty higher compared to the study by Rosediani et al. (2012) conducted in a single tertiary setting [26]. However, despite having good knowledge and attitude toward PFME, 54.8% of our respondents did not practice PFME during pregnancy. An explaination to this could be that respondents in primary care settings were not provided with proper intervention and also did not view their conditions seriously.

A recent study was conducted and found that intervention using two sessions of exercise education classes, improved PFME practices among pregnant women [18]. The study increased the number of multiparous women practicing PFME from only 5.8% before the intervention to 37.2% after the intervention [18]. Three factors that contributed tp low PFME practices were; lack of PFME promotion by medical staffs during antenatal checkups, the pregnant women never had discussion with friends on PFME with friends and they did not seek PFME information [18].

The importance of PFME practices is that they can prevent several long-term complications in future for example severe UI, faecal incontinence and in the worst case pelvic organ prolapse [38–40].

### Limitation of this study

This study was a cross-sectional study which temporal relationship cannot be determined to elicit the cause and effect of the disease. Also being a single centred study, the findings cannot be representative or generalised to a larger population.

## Conclusion

This study highlighted that almost half of the respondents had UI, which occurred even from the first trimester. Pregnant women who were obese and multiparous were at high risk to have UI. PFME practices were low despite remarkable good scores in attitude knowledge. This study emphasizes the importance of correct knowledge transfer to ensure good PFME practices among pregnant women.

## Supporting information

S1 Data (XLSX)
